# Optimizing nitrogen and phosphorus application to improve soil organic carbon and alfalfa hay yield in alfalfa fields

**DOI:** 10.3389/fpls.2023.1276580

**Published:** 2024-01-19

**Authors:** Kongqin Wei, Junwei Zhao, Yanliang Sun, Ignacio F. López, Chunhui Ma, Qianbing Zhang

**Affiliations:** ^1^ College of Animal Science and Technology, Shihezi University, Shihezi, Xinjiang, China; ^2^ School of Agriculture and Environment, Massey University, Palmerston North, New Zealand

**Keywords:** nitrogen fertilizer, phosphorus fertilizer, soil organic carbon, soil reactive organic carbon, alfalfa, hay yield

## Abstract

Soil organic carbon (SOC) is the principal factor contributing to enhanced soil fertility and also functions as the major carbon sink within terrestrial ecosystems. Applying fertilizer is a crucial agricultural practice that enhances SOC and promotes crop yields. Nevertheless, the response of SOC, active organic carbon fraction and hay yield to nitrogen and phosphorus application is still unclear. The objective of this study was to investigate the impact of nitrogen-phosphorus interactions on SOC, active organic carbon fractions and hay yield in alfalfa fields. A two-factor randomized group design was employed in this study, with two nitrogen levels of 0 kg·ha^-1^ (N_0_) and 120 kg·ha^-1^ (N_1_) and four phosphorus levels of 0 kg·ha^-1^ (P_0_), 50 kg·ha^-1^ (P_1_), 100 kg·ha^-1^ (P_2_) and 150 kg·ha^-1^ (P_3_). The results showed that the nitrogen and phosphorus treatments increased SOC, easily oxidized organic carbon (EOC), dissolved organic carbon (DOC), particulate organic carbon (POC), microbial biomass carbon (MBC) and hay yield in alfalfa fields, and increased with the duration of fertilizer application, reaching a maximum under N_1_P_2_ or N_1_P_3_ treatments. The increases in SOC, EOC, DOC, POC, MBC content and hay yield in the 0–60 cm soil layer of the alfalfa field were 9.11%-21.85%, 1.07%-25.01%, 6.94%-22.03%, 10.36%-44.15%, 26.46%-62.61% and 5.51%-23.25% for the nitrogen and phosphorus treatments, respectively. The vertical distribution of SOC, EOC, DOC and POC contents under all nitrogen and phosphorus treatments was highest in the 0–20 cm soil layer and tended to decrease with increasing depth of the soil layer. The MBC content was highest in the 10–30 cm soil layer. DOC/SOC, MBC/SOC (excluding N_0_P_1_ treatment) and POC/SOC were all higher in the 0–40 cm soil layer of the alfalfa field compared to the N_0_P_0_ treatment, indicating that the nitrogen and phosphorus treatments effectively improved soil fertility, while EOC/SOC and DOC/SOC were both lower in the 40–60 cm soil layer than in the N_0_P_0_ treatment, indicating that the nitrogen and phosphorus treatments improved soil carbon sequestration potential. The soil layer between 0-30 cm exhibited the highest sensitivity index for MBC, whereas the soil layer between 30-60 cm had the highest sensitivity index for POC. This suggests that the indication for changes in SOC due to nitrogen and phosphorus treatment shifted from MBC to POC as the soil depth increased. Meanwhile, except the 20–30 cm layer of soil in the N_0_P_1_ treatment and the 20–50 cm layer in the N_1_P_0_ treatment, all fertilizers enhanced the soil Carbon management index (CMI) to varying degrees. Structural equation modeling shows that nitrogen and phosphorus indirectly affect SOC content by changing the content of the active organic carbon fraction, and that SOC is primarily impacted by POC and MBC. The comprehensive assessment indicated that the N_1_P_2_ treatment was the optimal fertilizer application pattern. In summary, the nitrogen and phosphorus treatments improved soil fertility in the 0–40 cm soil layer and soil carbon sequestration potential in the 40–60 cm soil layer of alfalfa fields. In agroecosystems, a recommended application rate of 120 kg·ha^-1^ for nitrogen and 100 kg·ha^-1^ for phosphorus is the most effective in increasing SOC content, soil carbon pool potential and alfalfa hay yield.

## 1 Introduction

Alfalfa (*Medicago sativa* L.) is a high quality perennial legume forage grass, known as the “king of forage grasses”, which not only supports the development of animal husbandry but also plays an important role in ecological restoration and soil quality improvement (Zhang et al., 2009; Li et al., 2019). Nitrogen and phosphorus are the most important mineral elements limiting the growth and development of alfalfa (Gren et al., 2012; Yuan et al., 2020). In field management, fertilizer application is often used to improve alfalfa hay yield and utilization years, but imbalanced fertilization has resulted in lower alfalfa yields and reduced soil quality (Li et al., 2015). Soil organic carbon (SOC) content is not only an important indicator of soil health and fertility, but also plays a dual role as a carbon source and sink in the soil carbon cycle, which is important to the carbon balance of farmland ecosystems and the sustainable and stable use of farmland soils. Long-term inputs of nitrogen and phosphorus have been shown to increase the level of SOC content in alfalfa fields (Gu et al., 2023). Additionally, it has been shown that when nitrogen and phosphorus are applied in the establishment year only, the SOC content of alfalfa fields increases significantly in the second year. However, by the eighth year, the SOC content has decreased to a minimal level. (Wang et al., 2020). Furthermore, high nutrient inputs can impact the production performance of alfalfa (Wang et al., 2023). Therefore, it is necessary to establish appropriate fertilizer application methods to enhance the SOC content and hay yield in alfalfa fields.

The dynamics of SOC are influenced by several factors, including the quantity of nitrogen and phosphorus supplied (Li et al., 2022), the manner of fertilizer application (Govednik et al., 2023) and the type of soil (Yuan et al., 2022). Prior research has indicated that the use of nitrogen has a positive influence on SOC levels and soil fertility (Li et al., 2023). It has been observed that the application of nitrogen fertilizer leads to soil acidification, which hinders the breakdown process of organic carbon. However, it concurrently enhances the stability of SOC (Zeng et al., 2023). Nitrogen application promotes plant carbon input processes and increases SOC accumulation (Xu et al., 2021). The application of nitrogen also induces alterations in the composition of soil microbial communities, hence facilitating the build up of SOC (Tang et al., 2023). Concurrently, the utilization of nitrogen and phosphorus increased the levels of soil nutrients (with initial soil phosphorus levels of 0.45 g·kg^-1^ and 0.76 g·kg^-1^), which led to the promotion of alfalfa growth and an increase in photosynthetic rates, consequently leading to elevated inputs of soil carbon (Hakl et al., 2021; Sun et al., 2022; Wang et al., 2023). It has been demonstrated that the application of phosphorus has an impact on the content of SOC, but not alter the geographical distribution of SOC fractions. (Hu et al., 2022). In contrast to the application of individual nutrients, the addition of nitrogen and phosphorus not only enhanced soil acidity but also mitigated the nitrogen and phosphorus deficiencies induced by single nutrient inputs. This combined approach had a beneficial synergistic impact on the build up of SOC (Tie et al., 2022). Previous research has demonstrated that the application of nitrogen and phosphorus has distinct effects on SOC content through separate mechanisms. Specifically, nitrogen input facilitates the input of carbon from litter matter, while phosphorus input hinders the decomposition of organic carbon. These processes synergistically contribute to the overall enhancement of SOC sequestration (Zhang et al., 2022a). However, it has also been found that the combination of nitrogen and phosphorus applications enhanced the abundance of SOC degradation genes and promoted the decomposition of SOC (Ye et al., 2023). Nitrogen and phosphorus additions also affect the stoichiometric ratios of carbon, nitrogen, and phosphorus in the soil, as well as the nutrient balance of soil microorganisms. These changes modify the decomposition of SOC by altering the intensity of the priming effect (Qin et al., 2024). Furthermore, the influence of co-applying nitrogen and phosphorus fertilizers on the amounts of SOC in various soil strata exhibits variability. Study has demonstrated that the concurrent utilization of nitrogen and phosphorus fertilizers leads to augment the proportion of SOC in both surface and deep soil layers (Xu et al., 2021). Nevertheless, research has also indicated that employing this approach results in a reduction in SOC content inside the uppermost layer. The decrease in carbon stocks can be ascribed to the rise in microbial biomass and the heightened functioning of carbon-degrading enzymes (Luo et al., 2019). In conclusion, the findings pertaining to the impact of nutrient inputs on SOC sequestration exhibit variability, with a predominant emphasis in prior research on the influence of nutrient inputs on organic carbon levels specifically within the surface soil layer. Previous research has indicated that subsoil has higher carbon stocks than Surface soil (Button et al., 2022), little is known about the changes in subsoil organic carbon due to nitrogen and phosphorus inputs. Therefore, this experiment investigated the changes in SOC content in the 0–60 cm soil layer of an alfalfa field as a result of nitrogen and phosphorus inputs.

SOC may be categorized into active and inert organic carbon based on its stability, where soil active organic carbon is sensitive to fertilizer management and can be a better indicator of early changes in soil quality than SOC (Li et al., 2021c). Soil active organic carbon includes soil microbial biomass carbon (MBC), easily oxidized organic carbon (EOC), dissolved organic carbon (DOC) and particulate organic carbon (POC) (Chen et al., 2016). Study has demonstrated that the application of nitrogen and phosphorus has the capacity to alter the carbon source preferences of soil microorganisms, resulting in an increase in the concentration of active organic carbon. (Li et al., 2021a). However, it has also been found that the use of fertilizer leads to an augmentation in nutrient turnover and expedites the depletion of active organic carbon in the soil (Shi et al., 2022). Previous researchers have developed a soil carbon pool management index (CMI) to assess changes in SOC and reactive organic carbon (Blair et al., 1995). The findings of this study indicate that the CMI exhibits a significant degree of sensitivity to various agricultural land management strategies (Zhu et al., 2015). Hence, it is important to comprehend the alterations in the active organic component of soil and the CMI resulting from various nitrogen and phosphorus treatments. This understanding has significance in the context of organic carbon sequestration and the pursuit of sustainable development in agricultural soils.

The arid region in Northwest China is expansive and ecologically delicate, containing a substantial amount of stored organic carbon. Nevertheless, recent research indicates a decline in the region’s SOC content over the last three decades, resulting in diminished soil fertility and crop yields (Zhang et al., 2022b). Therefore, our objective was to establish a fertilization regime that promotes alfalfa hay production and improves SOC sequestration. A 2-year nutrient addition trial was conducted in Shihezi, Xinjiang to investigate (1) the response of SOC and active organic carbon fractions to nitrogen and phosphorus inputs in 0–60 cm soils of alfalfa fields; (2) the contribution of soil active organic carbon fractions to SOC accumulation; and (3) the relationship between alfalfa hay yield, SOC and soil active organic carbon fractions under nitrogen and phosphorus fertilizer supplementation.

## 2 Materials and methods

### 2.1 Experimental site

The experimental site is located at the Shihezi University Water-saving Irrigation Experimental Station in Shihezi, Xinjiang (44°20′ N, 88°30′ E), which belongs to the temperate continental arid climate. The average temperature during the growing season of alfalfa is 25°C, the average annual temperature is 8°C, the average annual sunshine duration is 2770 h, the annual frost-free period is 168–171 d, the annual precipitation is 113–170 mm, and the average precipitation during the growing season of alfalfa is 50 mm (mostly concentrated from June to late August). The soil type of the test field is gray desert soil with the following physical and chemical properties: total nitrogen 1.18 g·kg^-1^, alkaline nitrogen 145.47 mg·kg^-1^, total phosphorus 0.53 g·kg^-1^, available phosphorus 19.30 mg·kg^-1^, available potassium 119.80 mg·kg^-1^, organic matter 21.56 g·kg^-1^, bulk weight 1.54 g·cm^-3^, pH=7.26.

### 2.2 Experimental design and crop management

The experiment was conducted in a two-factor randomized group design with two levels of nitrogen application: 0 kg·ha^-1^ (N_0_) and 120 kg·ha^-1^ (N_1_), and four levels of phosphorus application: 0 kg·ha^-1^ (P_0_), 50 kg·ha^-1^ (P_1_), 100 kg·ha^-1^ (P_2_) and 150 kg·ha^-1^ (P_3_), respectively. The determination of nitrogen and phosphorus fertilizer inputs was conducted using a field survey. The survey involved selecting fertilizer application rates (N_1_ and P_2_) that are commonly used in high-yielding local alfalfa fields. These rates were then adjusted by increasing and decreasing them by 50% to establish the appropriate fertilizer application rate. Alfalfa, a leguminous pasture, naturally produces rhizomes that provide nitrogen to the plant (Lagunas et al., 2019). Meanwhile, phosphorus application enhances the development of efficient rhizomes and boosts nitrogen-fixing enzyme activity, resulting in increased fixation of free nitrogen by rhizomes (Schulze et al., 2011). Studies have shown that applying nitrogen does not improve alfalfa yield and instead reduces the plant’s nitrogen fixation capacity (Xie et al., 2015). Therefore, only two levels of nitrogen fertilizer were considered necessary. Fertilizer was applied in drips with water 3–5 days after mowing the first, second and third crops. The application of fertilizer occurred on the following dates: 19 April, 25 May, 4 July, and 14 August 2020, as well as 18 April, 27 May, 3 July, and 4 August 2021.

The test alfalfa variety was WL366HQ, which was manually sown in strips on 29 April 2019 with a row spacing of 20 cm, a sowing depth of 2 cm and a sowing rate of 18.0 kg·ha^-1^. A 1 m wide pedestrian passage was set up between each plot to effectively isolate the fertilizer and water from each other between the plots. The trial employed subsurface drip irrigation as the chosen irrigation method. The drip tape was situated at a height of 10 cm above the ground, aligned with the direction of the alfalfa strips. The drip heads were evenly distributed at a spacing of 20 cm, while each drip strip was positioned at a spacing of 60 cm. The management practices in the field were mostly identical across all plots, with the exception of varying degrees of fertilizer application.

### 2.3 Sampling and measurements

#### 2.3.1 Soil sample collection

On 30th September 2020 and 24th September 2021, soil samples were collected from the same layer of soil in each plot at 6 different soil depths (0–10 cm, 10–20 cm, 20–30 cm, 30–40 cm, 40–50 cm, 50–60 cm) using a 3 cm diameter soil auger in accordance with the “five-point sampling method”, and the same layer of soil in each plot was mixed evenly and divided into 2 parts to remove visible dead branches and gravel by hand, and then further removed the root system by using a 2 mm sieve. One portion was placed in a self-sealing bag and immediately stored at a low temperature for the determination of MBC and DOC; the other portion was brought back to the laboratory for air drying and used for the determination of SOC, POC and EOC.

#### 2.3.2 Soil sample measurement

SOC content was determined using the potassium dichromate volumetric method with external heating; DOC was determined using the potassium dichromate method; MBC was determined using the chloroform fumigation-leaching method; EOC was determined using the KMnO_4_ oxidation method; and POC was determined using the sodium hexametaphosphate separation method (Lu, 2000; Jiang et al., 2006; Vieira et al., 2007).

#### 2.3.3 Hay yield measurement

Using the sample method, alfalfa plants of uniform growth were selected at the first flowering stage (around 5% of flowering), and alfalfa plants (1 m × 1 m) within the sample square (leaving a stubble height of 5 cm) were cut with scissors, weighed and recorded, and repeated three times. Three samples of 300 g of fresh alfalfa were taken back to the laboratory. Oven dried at 105°C for 30 min and then dried at 65°C to a constant weight to calculate the moisture content of the alfalfa and further convert the alfalfa dry matter yield.

#### 2.3.4 Soil reactive organic carbon efficiency and sensitivity index

The equation for the effective rate of the soil active organic carbon fraction is (Yu et al., 2020):


$$\text{DOC efficiency rate}=\frac{\text{DOC content}}{\text{SOC content}}\times 100\%$$

$$\text{MBC efficiency rate}=\frac{\text{MBC content}}{\text{SOC content}}\times 100\%$$

$$\text{EOC efficiency rate}=\frac{\text{EOC content}}{\text{SOC content}}\times 100\%$$

$$\text{POC efficiency rate}=\frac{\text{POC content}}{\text{SOC content}}\times 100\%$$

Soil active organic carbon fraction sensitivity index was calculated as (Chaudhary et al., 2017):


$$\text{Sensitivity index}=\frac{\text{active organic carbon content}-\text{reference active organic carbon content}}{\text{reference active organic carbon content}}\times 100\%$$

Where the measured soil active organic carbon content under the N_0_P_0_ treatment is used as the reference active organic carbon content.

#### 2.3.5 Carbon management index

The CMI was calculated based on the methods by Blair et al. (1995). In this study, N_0_P_0_ was used as the reference when calculating the CMI. The calculation is as follows:


$$\text{L}=\frac{\text{content of labile SOC}}{\text{content of non—labile SOC}}$$

where L refers to the carbon pool activity. The labile SOC content is expressed by the soil EOC content, and the non-labile SOC content is quantified by subtracting the content of EOC from the total SOC.


$$\text{LI=}\frac{\text{L in treatment}}{\text{L in reference}}$$

where LI is the lability index.


$$\text{CPI=}\frac{\text{SOC content of treatment}}{\text{SOC content of reference}}$$

where CPI is the carbon pool index.


CMI=CPI×LI×100

### 2.4 Data analysis

Data were collated using Microsoft Excel 2021 and data were analyzed for significance using SPSS 26.0. A three-way ANOVA was used to analyze the effects of nitrogen level, phosphorus level and soil depth on SOC, MBC, DOC, EOC, POC, soil reactive organic carbon efficiency, sensitivity index and carbon management index. One-way ANOVA was used to determine the significance (*P*< 0.05) of different nitrogen application treatments at the same phosphorus level and different phosphorus application treatments at the same nitrogen level on the content of SOC, MBC, DOC, EOC and POC, plotted and correlated using Origin 2022. A structural equation model was fitted to the effects of nitrogen application level, phosphorus application level and soil depth on SOC, MBC, DOC, EOC and POC using Amos 24.0 software.

## 3 Results

### 3.1 Soil organic carbon content

**Figure 1:**
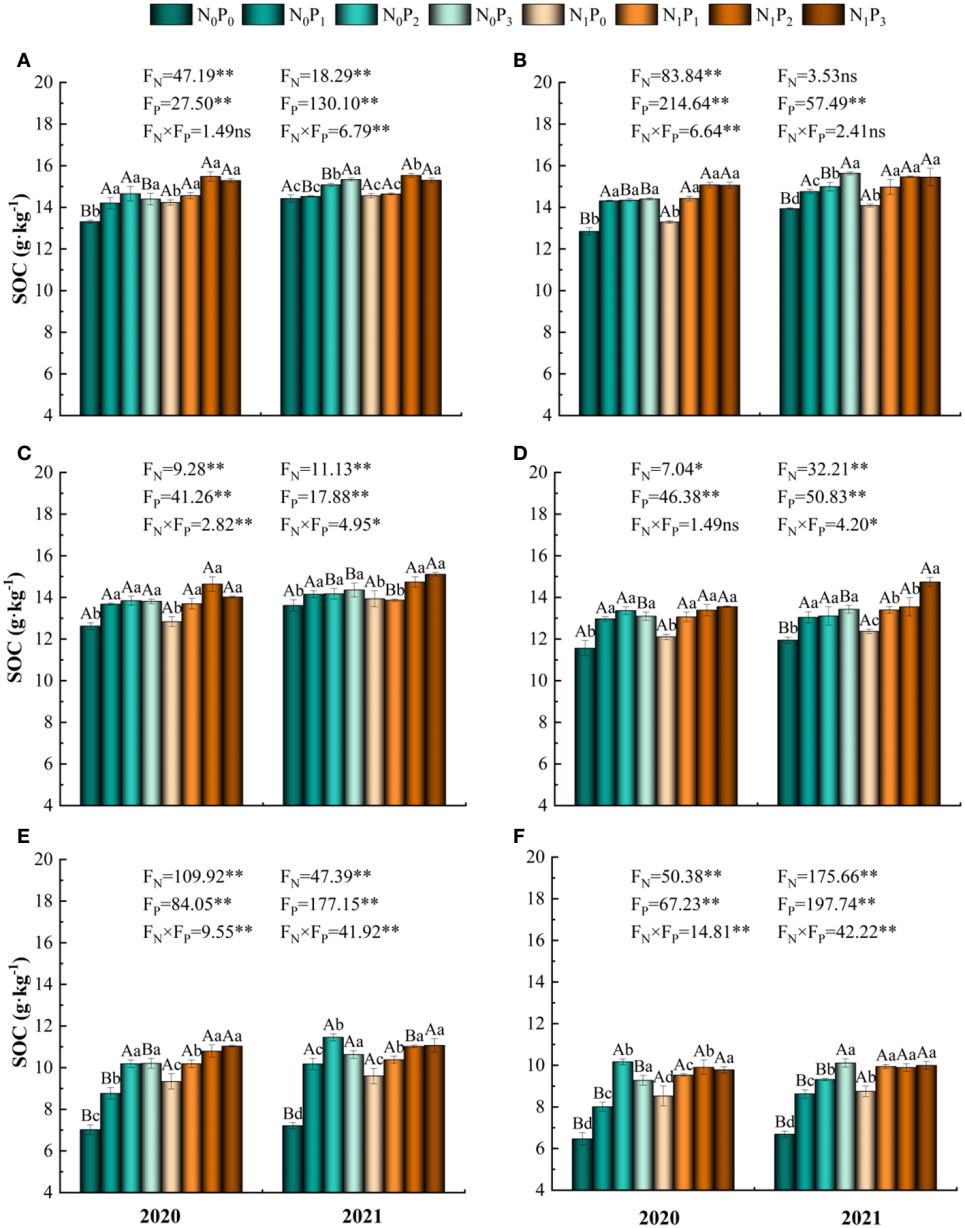
SOC content under different nitrogen and phosphorus treatments. Different lowercase letters indicate significant differences (*P*< 0.05) between different phosphorus application treatments at the same level of nitrogen application, and different capital letters indicate significant differences (*P*< 0.05) between different nitrogen application treatments at the same level of phosphorus application. * denotes *P*< 0.05, ** denotes *P*< 0.01 and ns denotes *P* > 0.05. **(A–F)** denote SOC content in the 0–10 cm, 10–20 cm, 20–30 cm, 30–40 cm, 40–50 cm and 50–60 cm soil layers, respectively.

**Figure 2:**
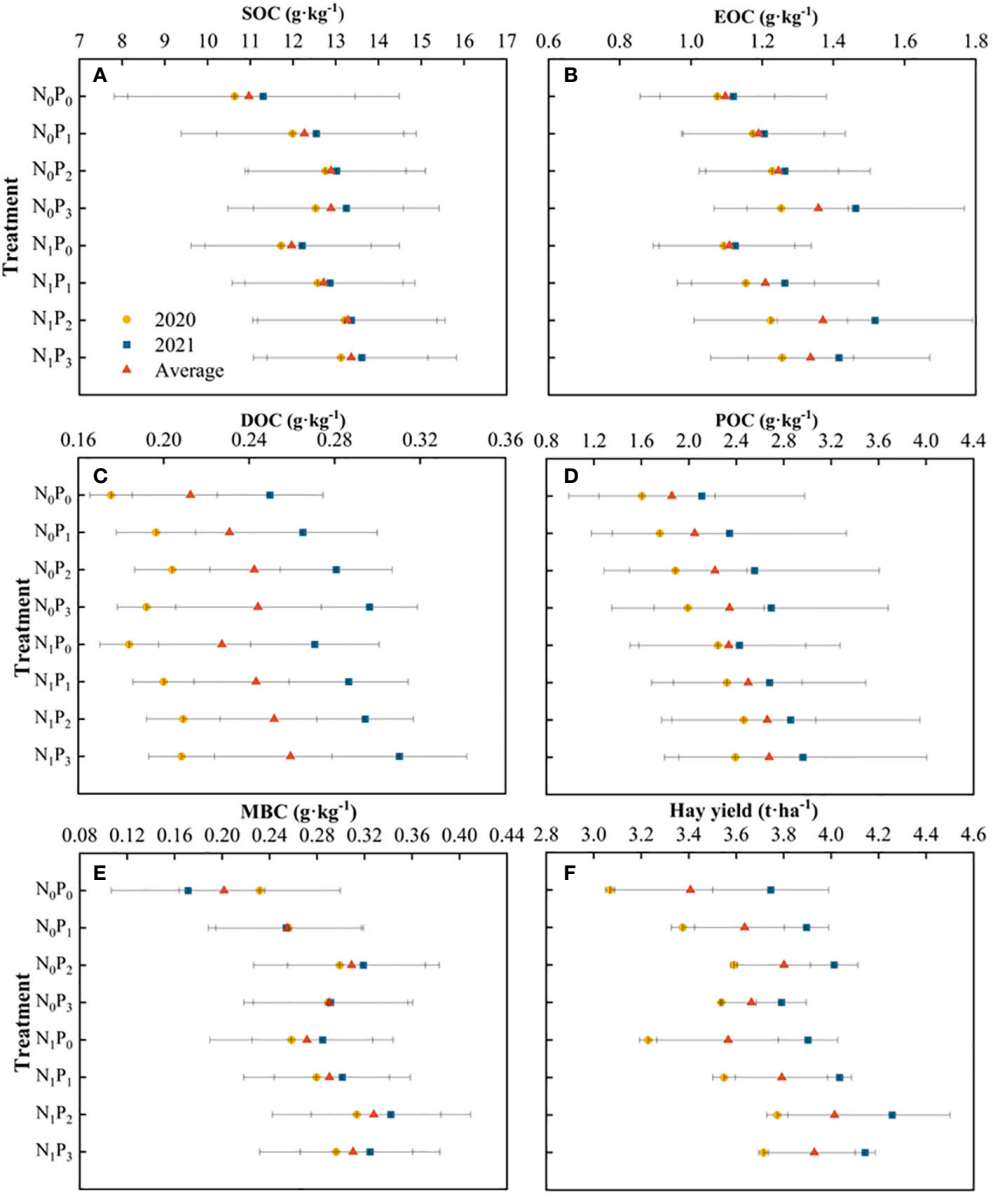
Soil organic carbon (SOC), active organic carbon fraction content (EOC、DOC、POC and MBC) and hay yield under different nitrogen and phosphorus treatments. **(A–F)** are represented separately SOC、EOC、DOC、POC、MBC and hay yield. SOC denotes soil organic carbon, EOC denotes readily oxidizable organic carbon, DOC denotes dissolved organic carbon, POC denotes particulate organic carbon, and MBC denotes quantity carbon.

The nitrogen and phosphorus treatments significantly increased SOC content and varied with soil depth (*P*< 0.05, **Table 1**; **Figure 1**). Compared to the N_0_P_0_ treatment, SOC content in the 0–60 cm soil layer increased by 0.70%-16.32%, 1.09%-17.38%, 1.86%-15.99%, 3.52%-23.42%, 24.89%-58.89% and 23.85%-57.23% under each nitrogen and phosphorus treatment, respectively (**Figure 1**; **Figure 2A**). SOC content was significantly greater under the P_2_ and P_3_ treatments than under the P_0_ treatment at the same level of nitrogen application (*P*< 0.05). Under nitrogen and phosphorus treatments, SOC content was greatest in 2020 under treatment N_1_P_2_ and highest in 2021 under treatment N_1_P_3_. Except for the 50–60 cm soil layer in 2020 and the 30–40 cm soil layer in 2021, the differences in SOC content between the N_1_P_2_ and N_1_P_3_ treatments were not significant (*P* > 0.05) (**Figure 1**). The SOC content in 2021 was higher than that in 2020 (**Figure 2A**).

**Table 1:** Analysis of variance (ANOVA) for the effect of nitrogen level, phosphorus level and soil depth on SOC and soil reactive organic carbon.

Variable	2020					2021				
	SOC	EOC	DOC	POC	MBC	SOC	EOC	DOC	POC	MBC
	(g·kg^-1^)	(g·kg^-1^)	(g·kg^-1^)	(g·kg^-1^)	(g·kg^-1^)	(g·kg^-1^)	(g·kg^-1^)	(g·kg^-1^)	(g·kg^-1^)	(g·kg^-1^)
Nitrogen(N)	238.94^**^	0.07^ns^	45.27^**^	4047.20^**^	206.80^**^	183.62^**^	293.40^**^	296.68^**^	1178.92^**^	1965.16^**^
Phosphoric(P)	345.51^**^	812.95^**^	86.49^**^	215.70^**^	480.03^**^	426.88^**^	1368.33^**^	322.68^**^	749.09^**^	1307.77^**^
Depth(D)	1982.02^**^	4056.18^**^	94.20^**^	4197.37^**^	2290.87^**^	3249.15^**^	3251.31^**^	554.72^**^	9074.16^**^	3607.97^**^
N×P	9.98^**^	8.76^**^	5.31^**^	35.75^**^	13.53^**^	15.91^**^	277.69^**^	4.47^**^	2.76^*^	69.43^**^
N×D	13.63^**^	100.09^**^	0.26^ns^	12.91^**^	5.96^**^	12.85^**^	5.18^**^	0.65^ns^	61.64^**^	63.33^**^
P×D	6.50^**^	12.32^**^	1.32^ns^	9.76^**^	17.08^**^	20.22^**^	12.10^**^	4.59^**^	37.72^**^	20.35^**^
N×P×D	6.57^**^	6.15^**^	0.78^ns^	14.73^**^	2.62^**^	15.67^**^	28.08^**^	4.50^**^	34.47^**^	19.36^**^

* denotes P< 0.05, ** denotes P< 0.01, ns denotes P > 0.05. Values in the table are ANOVA statistics (F values). N denotes nitrogen treatment, P denotes phosphorus treatment, D denotes soil depth, SOC denotes soil organic carbon, EOC denotes easily oxidized organic carbon, DOC denotes dissolved organic carbon, POC denotes particulate organic carbon, and MBC denotes microbial biomass carbon.

### 3.2 Soil reactive organic carbon fraction

#### 3.2.1 Soil easily oxidized organic carbon content

**Figure 3:**
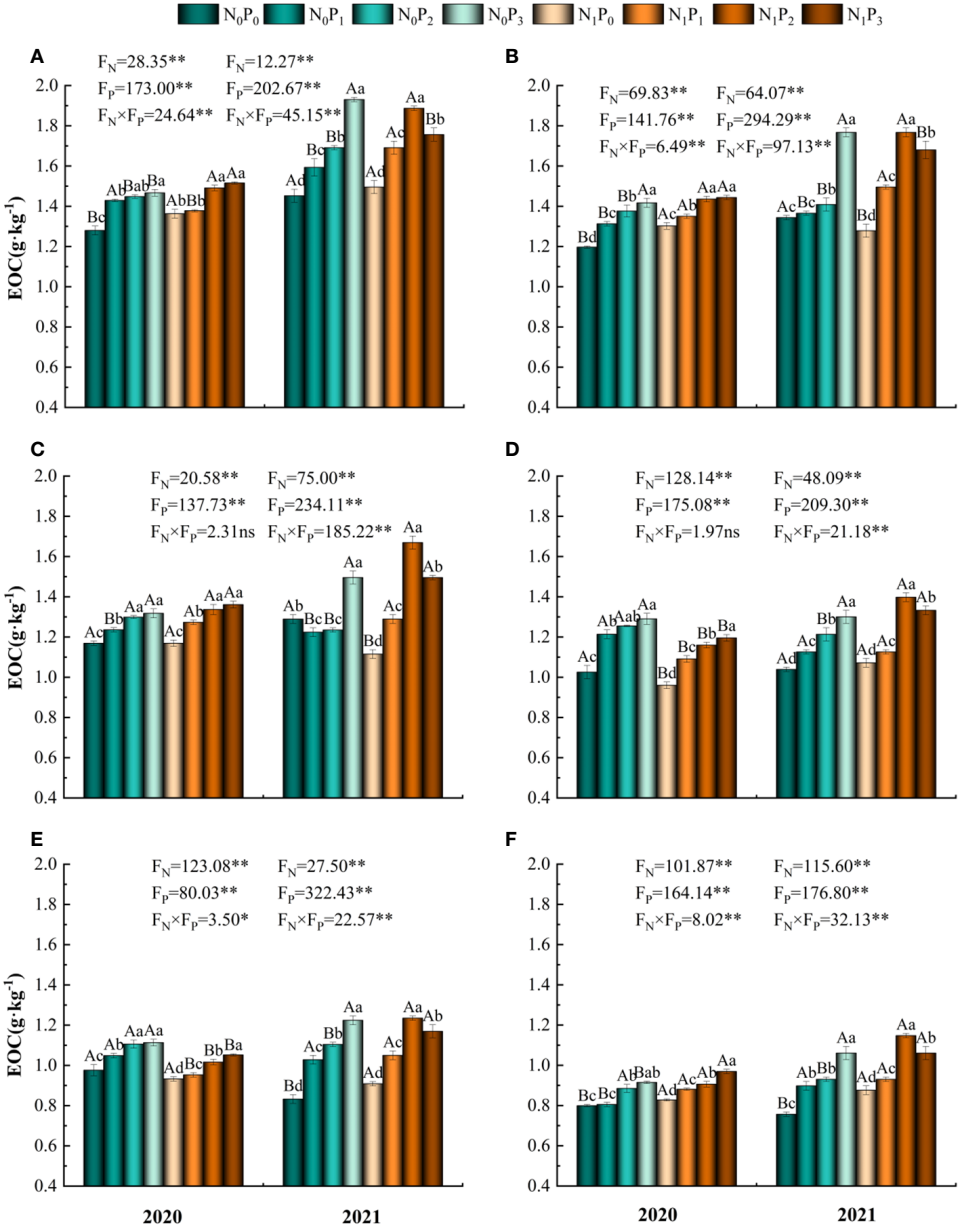
Soil easily oxidized organic carbon under different nitrogen and phosphorus treatments. **(A–F)** indicate the easily oxidized organic carbon content of soils in the 0–10 cm, 10–20 cm, 20–30 cm, 30–40 cm, 40–50 cm and 50–60 cm soil layers, respectively. * denotes *P* < 0.05, ** denotes *P* < 0.01 and ns denotes *P* > 0.05.

The content of EOC was significantly influenced by nitrogen and phosphorus treatments, soil depth and their interactions (*P*< 0.05, **Table 1**). In comparison to the N_0_P_0_ treatment, EOC content in the 0–60 cm soil layer increased by 2.99%-32.93%, -4.85%-31.55%, -13.49%-29.51%, -6.38%-35.52%, -4.45%-48.30% and 0.75%-51.72% under each nitrogen and phosphorus treatment, respectively. Under the N_0_ treatment, EOC content of the 0–60 cm soil layer tended to increase with increasing phosphorus application in 2020 and 2021 (except for the 20–30 cm soil layer in 2021), reaching a maximum under the P_3_ treatment and significantly greater than the P_0_ treatment (*P*< 0.05) (**Figure 3**). Under the N_1_ treatment, EOC content in the 0–60 cm soil layer tended to increase with increasing phosphorus application in 2020, reaching a maximum in the P_3_ treatment and significantly greater than in the P_1_ and P_0_ treatments (*P*< 0.05). The EOC content in all soil layers tended to increase and then decrease in 2021, reaching a maximum in the P_2_ treatment and being significantly greater than in the other three treatments (*P*< 0.05). At the same level of phosphorus application, the differences in EOC content between the N_0_ and N_1_ treatments were significant (except for the P_0_ treatment in 2021 and the P_3_ treatment in 2020) in the 0–10 cm and 10–20 cm soil layers (*P*< 0.05). EOC content was higher in 2021 than in 2020 and highest in N_1_P_2_ (**Figure 2B**).

#### 3.2.2 Soil dissolved organic carbon content

**Figure 4:**
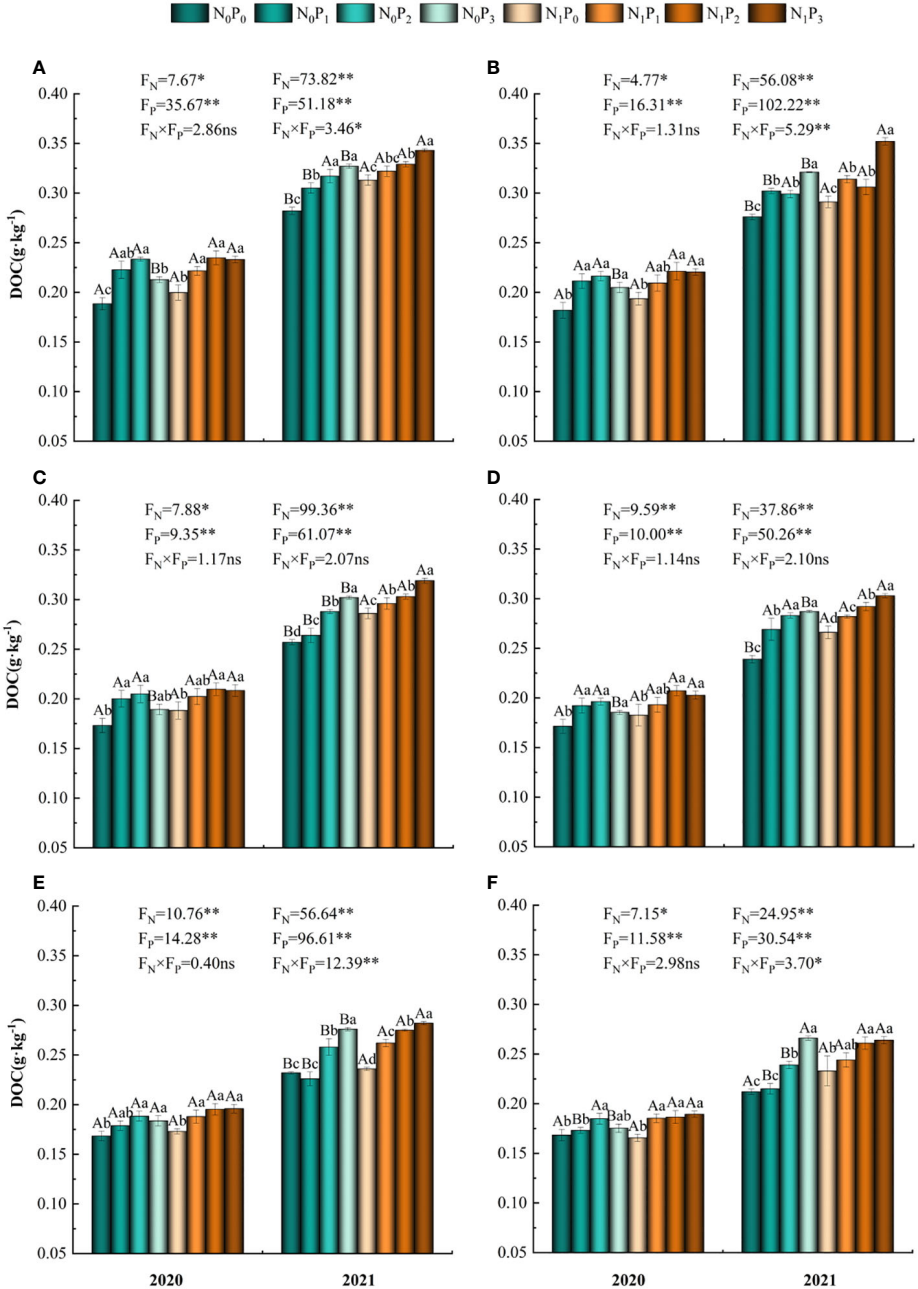
Soil dissolved organic carbon content under different nitrogen and phosphorus treatments. **(A-F)** indicate soil dissolved organic carbon content in the 0–10 cm, 10–20 cm, 20–30 cm, 30–40 cm, 40–50 cm and 50–60 cm soil layers, respectively. * denotes *P* < 0.05, ** denotes *P* < 0.01 and ns denotes *P* > 0.05.

The concentration of DOC was influenced by nitrogen and phosphorus treatments and soil depth (**Table 1**). In 2020, the application of phosphorus under the N_0_ treatment resulted in a pattern of rising and subsequently decreasing DOC content in each soil layer. The highest DOC content was seen under the P_2_ treatment, which was substantially larger than the P0 treatment (*P*< 0.05) (**Figure 4**). The phosphorus application treatment was significantly greater than the P_0_ treatment (*P*< 0.05). Furthermore, in the soil layer ranging from 40–60 cm, both the P_2_ and P_3_ treatments shown considerably higher values compared to the P_0_ treatment (*P*< 0.05). Under the N_1_ treatment, the P_2_ and P_3_ treatments significantly increased the soil DOC content of the alfalfa field in 2020 compared to the P_0_ treatment (*P*< 0.05), but the difference between the phosphorus treatments was not significant (*P* > 0.05). In 2021, DOC content tended to increase with increasing phosphorus application, reaching a maximum under the P_3_ treatment and in the 0–50 cm soil layer, the P_3_ treatment was significantly higher than the P_2_, P_1_ and P_0_ treatments (*P*< 0.05). Under the P_3_ treatment, DOC content was significantly higher (*P*< 0.05) in the N_1_ treatment than in the N_0_ treatment in the 0–40 cm and 50–60 cm soil layers in 2020, while the difference between the N_1_ and N_0_ treatments was not significant (*P* > 0.05) in the 40–50 cm soil layer. Under the other phosphorus treatments, the differences between the nitrogen application treatments were not significant (*P* > 0.05). In the 0–20 cm soil layer in 2021, the differences between N_0_ and N_1_ treatments were significant (*P<* 0.05), except for the P_2_ treatment where the differences between N_0_ and N_1_ treatments were not significant (*P* > 0.05), and in the 20–30 cm and 40–50 cm soil layers, the differences between N_0_ and N_1_ treatments were significant (*P*< 0.05) (**Figure 4**). The mean DOC content showed a trend of increasing, then decreasing and then increasing under the nitrogen and phosphorus treatments. Furthermore, the DOC concentration in 2021 surpassed that of 2020 (**Figure 2C**).

#### 3.2.3 Soil particulate organic carbon content

**Figure 5:**
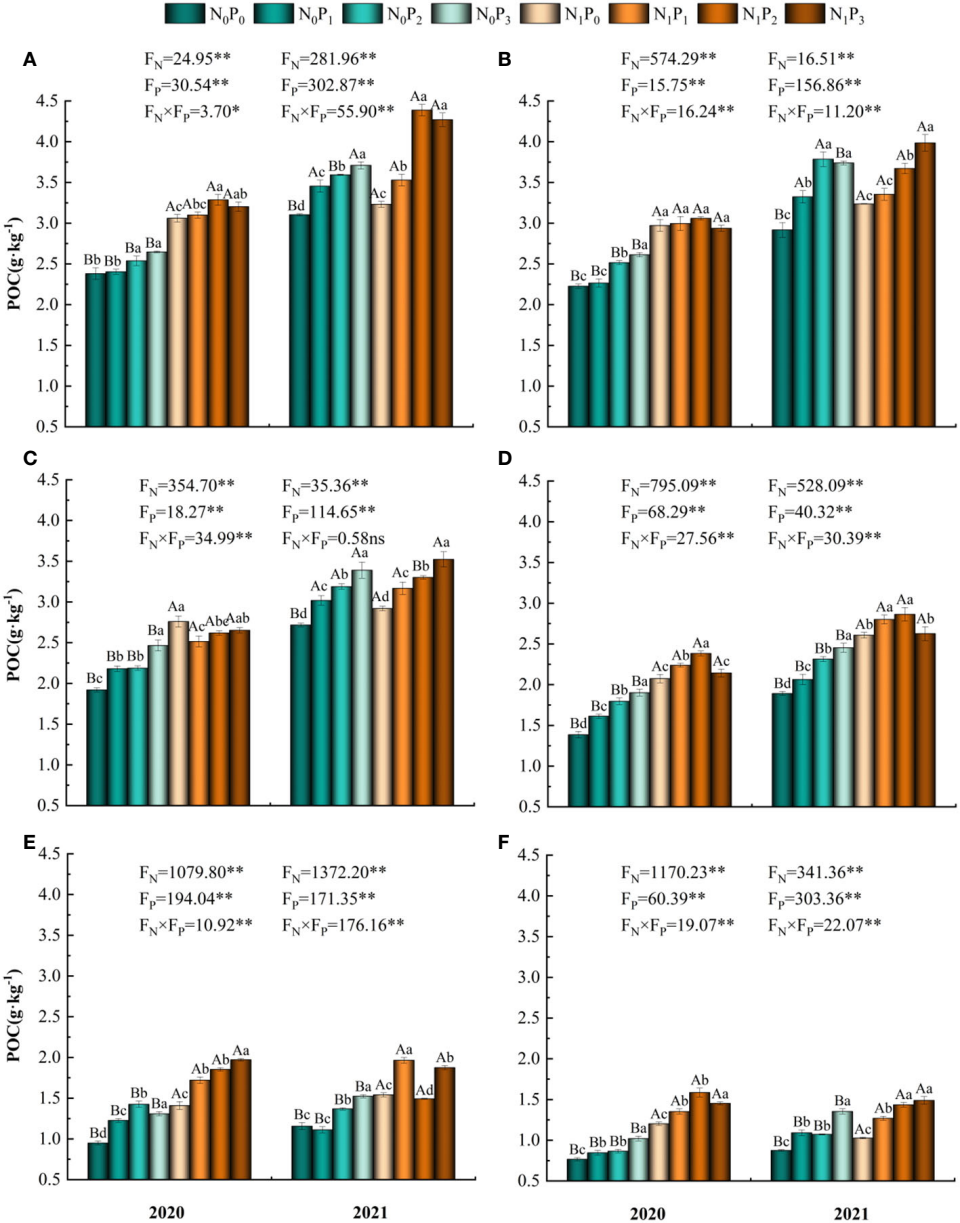
Particulate organic carbon content of soil under different nitrogen and phosphorus treatments. **(A-F)** indicate the particulate organic carbon content of soil in the 0–10 cm, 10–20 cm, 20–30 cm, 30–40 cm, 40–50 cm and 50–60 cm soil layers, respectively. * denotes *P* < 0.05, ** denotes *P* < 0.01 and ns denotes *P* > 0.05.

The POC content was substantially influenced by the application of nitrogen and phosphorus treatments, as well as the soil depth, and their interaction (*P*< 0.05, **Table 1**). In the N_0_ treatment, the levels of POC in the soil layers at depths of 10–20 cm and 40–50 cm exhibited a pattern of initial increase followed by decrease upon phosphorus application in both 2021 and 2020. The highest POC content was observed under the P_2_ treatment. Conversely, in the other soil layers, POC content showed a gradual increase with phosphorus application, reaching its peak under the P_3_ treatment (**Figure 5**). Under the N_0_ treatment, the content of POC in all soil layers was significantly higher under the P_2_ and P_3_ treatments than under the P_0_ treatment (*P*< 0.05). Under the N_1_ treatment, the content of POC in the 0–20 cm, 30–40 cm, 50–60 cm, 0–10 cm and 30–40 cm soil layers in 2020 tended to increase and then decrease with increasing phosphorus application in 2021, with the highest content under the P_2_ treatment. POC content in the 10–30 cm, 50–60 cm and 40–50 cm soil layers in 2021 and 2020 tended to increase gradually with increasing phosphorus application. Under the N_1_ treatment, POC content was significantly higher (*P*< 0.05) in all soil layers under the P_2_ and P_3_ treatments than under the P_0_ treatment, except for the 20–30 cm and 40–50 cm layers in 2020 and 2021. Under the same phosphorus application treatment, POC content was significantly higher (*P*< 0.05) in the 0–60 cm soil layer in 2020 and in the 30–60 cm soil layer in 2021 under treatment N_1_ than under treatment N_0_. POC content increased, then decreased and then increased again in both years under all nitrogen and phosphorus treatments and was higher in 2021 than in 2020 (**Figure 2D**).

#### 3.2.4 Soil microbial biomass carbon content

**Figure 6:**
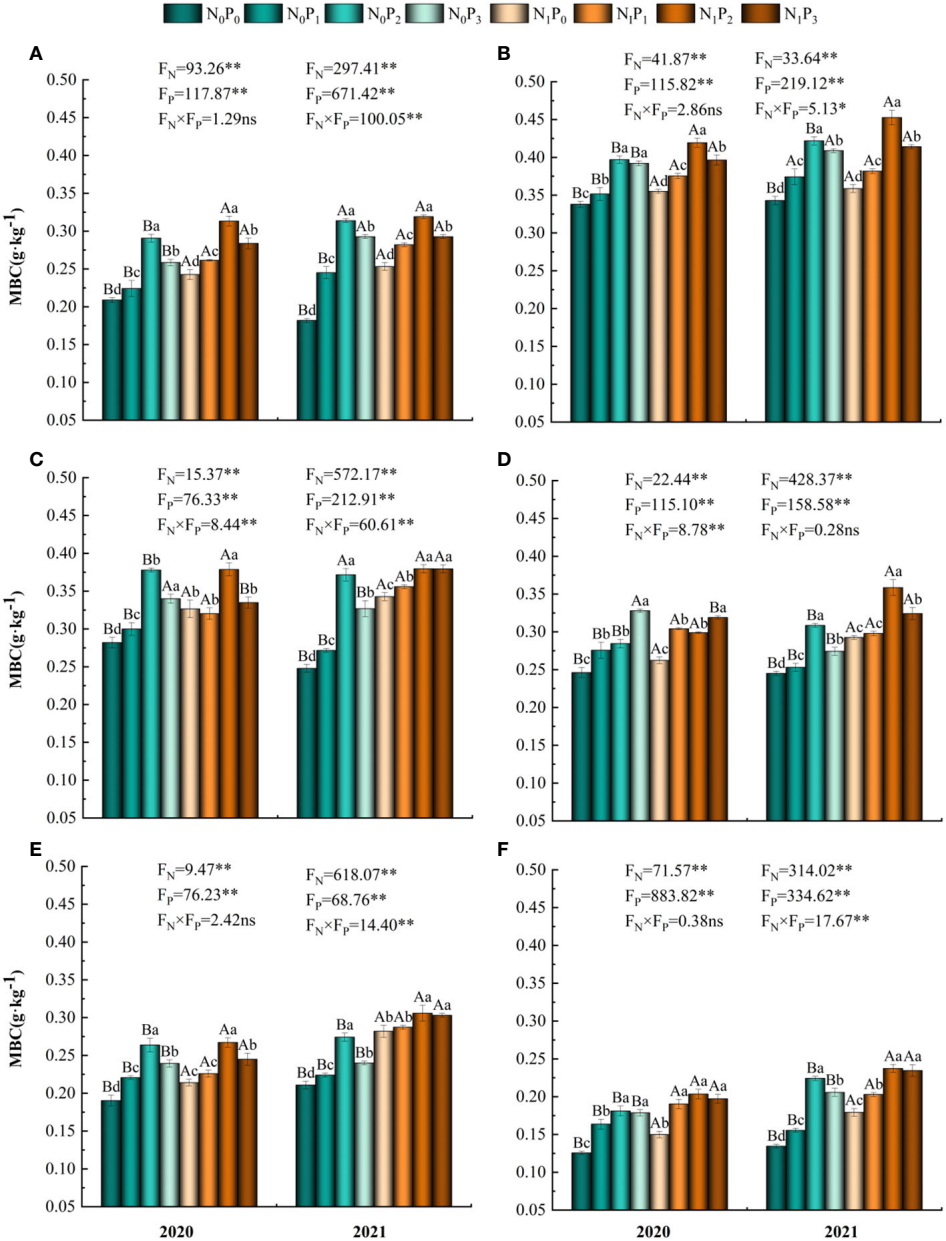
Soil microbial biomass carbon content under different nitrogen and phosphorus treatments. **(A–F)** indicate soil microbial carbon content in the 0–10 cm, 10–20 cm, 20–30 cm, 30–40 cm, 40–50 cm and 50–60 cm soil layers, respectively. * denotes *P* < 0.05, ** denotes *P* < 0.01 and ns denotes *P* > 0.05.

Nitrogen and phosphorus treatments, soil depth and their interactions significantly affected MBC content (*P*< 0.05, **Table 1**). Under the N_0_ and N_1_ treatments, MBC content in the 0–60 cm soil layer tended to increase and then decrease with increasing phosphorus application (except for the 30–40 cm soil layer in 2020), reaching a maximum under the P_2_ treatment, which was significantly greater than the P_0_ treatment (*P*< 0.05) (**Figure 6**). Under the same phosphorus application treatment, MBC content was significantly different between the N_1_ and N_0_ treatments in 2020 (*P*< 0.05) and was significantly higher in the N_1_ treatment than in the N_0_ treatment in the 30–60 cm soil layer in 2021 (*P*< 0.05). MBC content in all soil layers was highest in the 10–20 cm soil layer and lowest in the 50–60 cm soil layer. The MBC content tended to increase, then decrease, then increase and then decrease under the nitrogen and phosphorus treatments, with the highest content in the N_1_P_2_ treatment. Except for the N_0_P_0_ treatment, MBC content was higher in 2021 than in 2020 (**Figure 2E**).

### 3.3 Soil reactive organic carbon efficiency and sensitivity index

**Figure 7:**
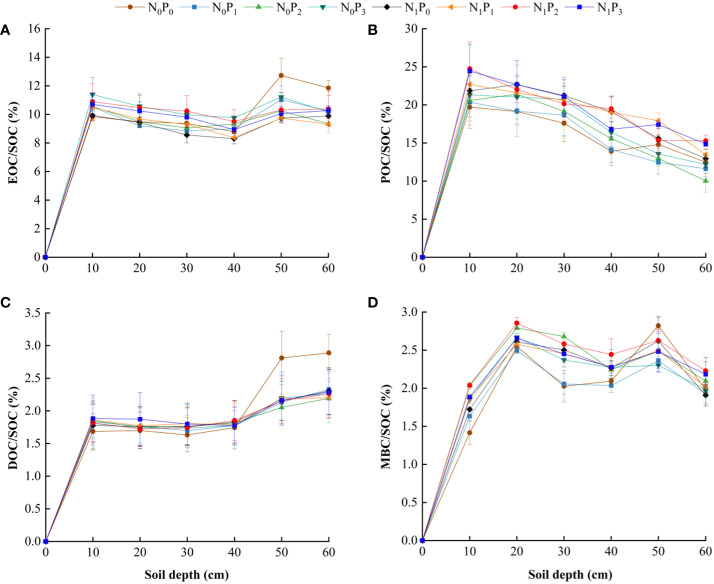
Effective rate of soil reactive organic carbon under different nitrogen and phosphorus treatments.

**Figure 8:**
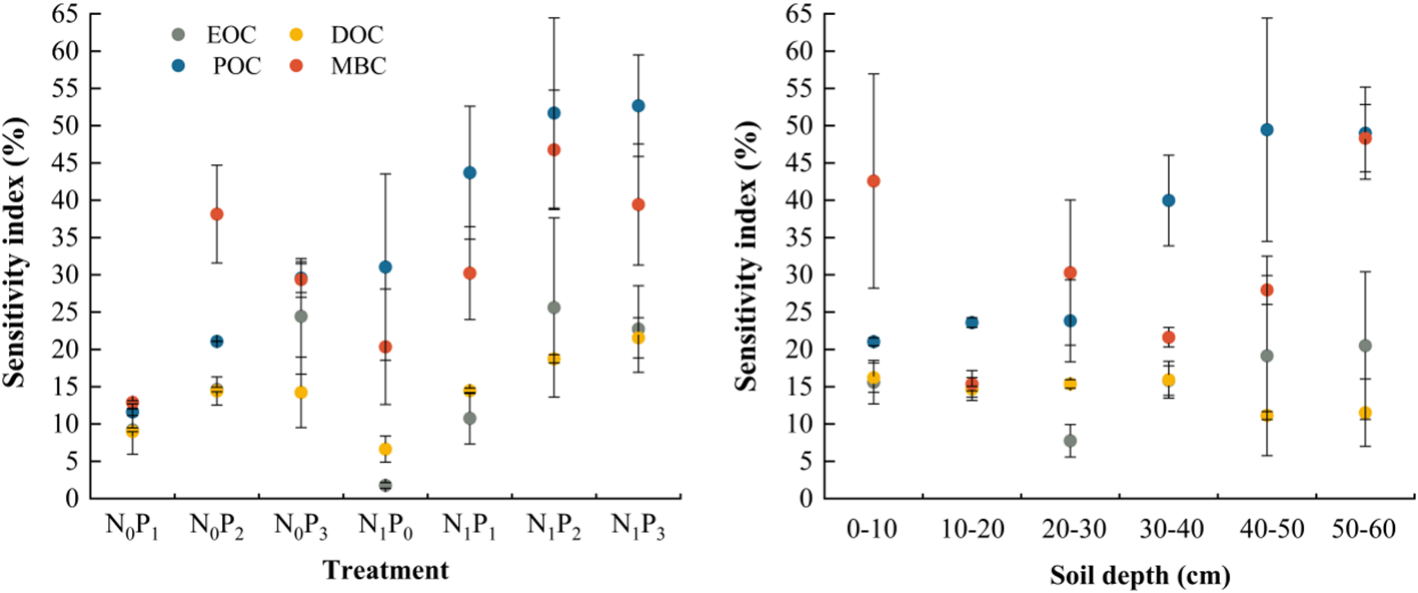
Sensitivity indices for soil reactive organic carbon under different nitrogen and phosphorus treatments. EOC indicates easily oxidized organic carbon, DOC indicates dissolved organic carbon, POC indicates particulate organic carbon and MBC indicates microbial biomass carbon.

Nitrogen application level, phosphorus application level and soil depth significantly affected the effective rate of soil reactive organic carbon (*P*< 0.05, **Table 2**), except for the non-significant effect of nitrogen application level on the effective rate of EOC in 2021 and MBC in 2020 (*P* > 0.05). Overall, EOC/SOC, DOC/SOC, POC/SOC and MBC/SOC were higher in the 0–40 cm soil layer than in the N_0_P_0_ treatment under all nitrogen and phosphorus treatments. In the 40–60 cm soil layer, EOC/SOC and DOC/SOC were lower under nitrogen and phosphorus treatments than under the N_0_P_0_ treatment (**Figure 7**). The highest MBC sensitivity index or POC sensitivity index was found under each nitrogen and phosphorus treatment. The soil layers that exhibited the highest sensitivity index for MBC were the 0–10 cm and 20–30 cm layers, whereas the soil layers with the highest sensitivity index for POC were the 10–20 cm and 30–60 cm levels (**Figure 8**).

**Table 2:** Analysis of variance (ANOVA) on the effect of nitrogen application level, phosphorus application level and soil depth on the efficiency of active organic carbon.

Variable	2020				2021			
	EOC/SOC	DOC/SOC	POC/SOC	MBC/SOC	EOC/SOC	DOC/SOC	POC/SOC	MBC/SOC
Nitrogen(N)	237.77^**^	18.69^**^	1601.15^**^	0.80^ns^	3.36^ns^	4.79^*^	414.13^**^	406.11^**^
Phosphoric(P)	26.21^**^	14.30^**^	6.90^**^	58.01^**^	165.03^**^	32.93^**^	82.64^**^	159.94^**^
Depth(D)	1125.91^**^	297.81^**^	580.83^**^	621.53^**^	246.08^**^	489.02^**^	1962.65^**^	495.30^**^
N×P	16.81^**^	15.38^**^	15.43^**^	3.29^*^	185.65^**^	22.74^**^	9.21^**^	15.42^**^
N×D	64.60^**^	13.91^**^	4.82^**^	20.04^**^	8.65^**^	14.58^**^	36.60^**^	18.71^**^
P×D	23.08^**^	11.10^**^	6.22^**^	19.22^**^	15.43^**^	25.71^**^	31.00^**^	31.30^**^
N×P×D	10.90^**^	6.95^**^	16.87^**^	9.15^**^	12.13^**^	24.07^**^	31.94^**^	13.44^**^

* indicates *P*< 0.05, ** indicates *P*< 0.01, ns indicates *P* > 0.05. Values in the table are ANOVA statistics (F values). N represents nitrogen treatment, P represents phosphorus treatment, D represents soil depth, SOC represents soil organic carbon, EOC represents easily oxidized organic carbon, DOC represents dissolved organic carbon, POC represents particulate organic carbon, and MBC represents microbial biomass carbon.

### 3.4 Carbon management index

**Figure 9:**
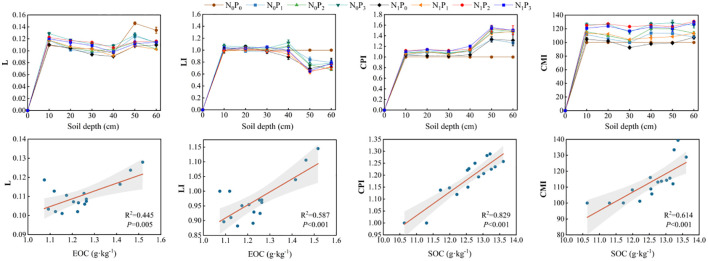
Soil carbon pool management index and correlation between carbon pool management index and EOC and SOC under different nitrogen and phosphorus treatments.

The carbon pool management index (CPI) was significantly affected by the rate of nitrogen application, phosphorus application, and soil depth (*P*< 0.05, **Table 3**). Nevertheless, the nitrogen application rate did not have a significant effect on L in 2021 (*P* > 0.05). The L and LI increased in the 0–10 cm soil layer but decreased in the 40–60 cm layer under all fertilizer treatments compared to the N_0_P_0_ treatment. The CPI was higher in the 0–60 cm soil layer under all fertilizer treatments than in the N_0_P_0_ treatment. Similarly, the CMI increased to varying degrees under all fertilizer treatments, except for N_0_P_1_ in the 20–30 cm layer and N_1_P_0_ in the 20–50 cm layer. The highest CMI was observed under N_1_P_2_ treatment. Correlation analysis revealed that the L and LI were significantly correlated with EOC, while the CPI and CMI were significantly correlated with SOC (**Figure 9**).

**Table 3:** Analysis of variance (ANOVA) on the effect of nitrogen application level, phosphorus application level and soil depth on the carbon management index.

Variable	2020				2021			
	L	LI	CPI	CMI	L	LI	CPI	CMI
Nitrogen(N)	239.25^**^	106.47^**^	69.23^**^	4.85^*^	3.49^ns^	3.98^*^	148.55^**^	136.62^**^
Phosphoric(P)	27.30^**^	10.42^**^	90.01^**^	240.10^**^	165.01^**^	126.23^**^	316.17^**^	619.70^**^
Depth(D)	126.17^**^	292.81^**^	160.50^**^	67.61^**^	245.80^**^	183.73^**^	940.30^**^	135.81^**^
N×P	17.97^**^	5.67^**^	4.87^**^	1.66^ns^	187.14^**^	130.64^**^	22.23^**^	128.87^**^
N×D	66.27^**^	23.41^**^	8.85^**^	41.79^**^	8.76^**^	5.88^**^	21.63^**^	4.82^**^
P×D	23.57^**^	10.55^**^	6.58^**^	5.71^**^	15.62^**^	11.98^**^	35.94^**^	5.14^**^
N×P×D	11.43^**^	4.74^**^	2.69^**^	2.16^*^	12.05^**^	8.11^**^	16.07^**^	8.42^**^

* denotes *P* < 0.05, ** denotes *P* < 0.01 and ns denotes *P* > 0.05. L represents the carbon pool activity, LI represents the lability index, CPI represents the carbon pool index, CMI represents carbon management index.

### 3.5 Structural equation modeling analysis

**Figure 10:**
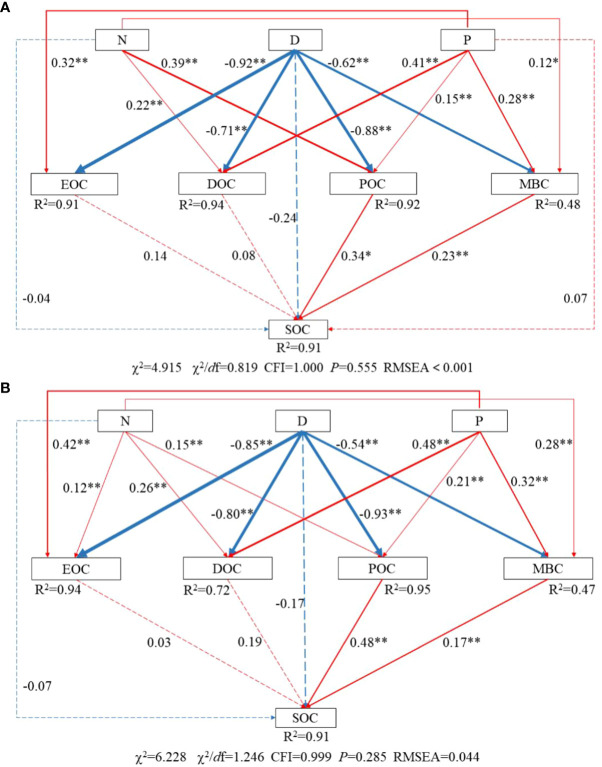
Structural equation modeling of the effect of nitrogen and phosphorus treatments on SOC and active organic carbon. **(A)** 2020, **(B)** 2021. The numbers next to the lines are normalized path coefficients, and the thickness of the lines indicates the size of the path coefficients. Red indicates a positive correlation, blue indicates a negative correlation, dashed lines indicate non-significant, and solid lines indicate significant. * indicates *P*< 0.05; ** indicates *P*< 0.01.

The structural equation model shows that the effect of nitrogen and phosphorus on SOC is mainly indirect, and the content of POC and MBC in different soil layers are the main factors affecting its change (**Figure 10**). In particular, there was no significant effect of nitrogen application on EOC in 2020 (*P* > 0.05), a result consistent with the analysis of variance results. Compared to 2020, the effect of POC content on SOC content increased in 2021, from 0.34 to 0.48, but reduced the effect of MBC content on SOC content, from 0.23 to 0.17.

### 3.6 Correlation analysis of alfalfa hay yield and measurement indicators

**Figure 11:**
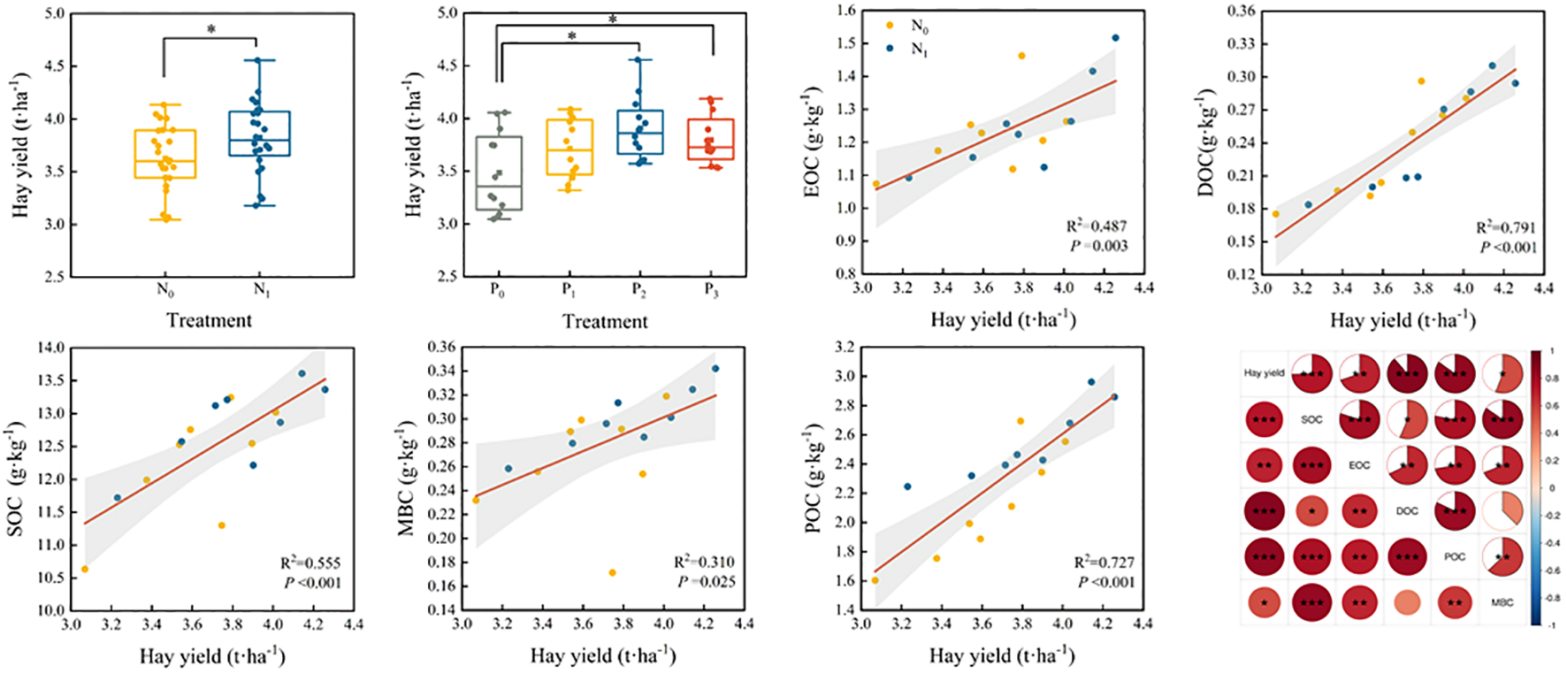
Effect of fertilization on hay yield and correlation analysis between hay yield and SOC, DOC, EOC, POC and MBC with hay yield. * indicates a significant level of *P<* 0.05, ** indicates a significant level of *P<* 0.01, *** indicates a significant level of *P<* 0.001.

Nitrogen and phosphorus inputs increased hay yield in alfalfa, with the highest hay yield under the N_1_P_2_ treatment (**Figure 2F**). Compared to the N_0_P_0_ treatment, alfalfa hay yield increased by 5.51%, 10.12%, 6.68%, 9.36%, 12.29%, 23.23% and 23.25% under each treatment, respectively (**Figure 11**). Regression analysis showed that SOC (R^2^ = 0.555), DOC (R^2^ = 0.791), EOC (R^2^ = 0.487), POC (R^2^ = 0.727) and MBC (R^2^ = 0.310) were each significantly correlated with hay yield (**Figure 11**).

### 3.7 Principal component analysis and comprehensive evaluation of measurement indicators

**Figure 12:**
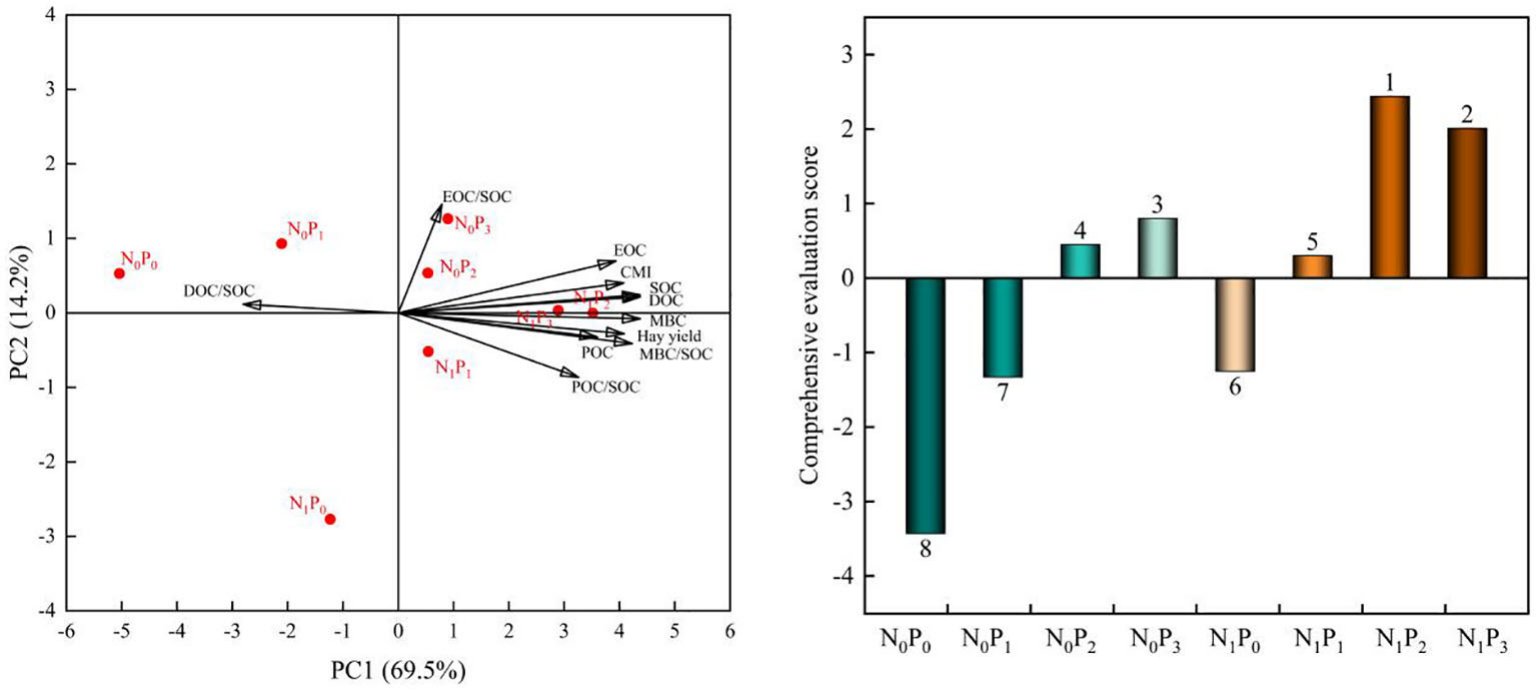
Principal component analysis and comprehensive evaluation of the measured indicators under nitrogen and phosphorus treatment. Principal component analysis showed that the eigenvalues of SOC (PC1 = 0.350, PC2 = 0.122) and DOC (PC1 = 0.350, PC2 = 0.108) were similar, so leading to the overlap of the two arrows.

SOC, active organic carbon fractions, CMI and alfalfa hay yield under nitrogen and phosphorus treatments were comprehensively evaluated by principal component analysis, and two principal components with eigenvalues greater than one were extracted, with a cumulative contribution of 83.7%. The principal component analysis showed that the combined scores of each nitrogen and phosphorus treatment were N_1_P_2_ (2.44) > N_1_P_3_ (2.01) > N_0_P_3_ (0.80) > N_0_P_2_ (0.45) > N_1_P_1_ (0.30) > N_1_P_0_ (-1.25) > N_0_P_1_ (-1.33) > N_0_P_0_ (-3.43) in order (**Figure 12**). Therefore, the N_1_P_2_ treatment was the optimal treatment to improve soil carbon sequestration and alfalfa hay production in alfalfa fields.

## 4 Discussion

### 4.1 Effect of nitrogen and phosphorus application on soil organic carbon and alfalfa hay yield in alfalfa field*s*

The primary contributor to SOC is the carbon inputs from plants, both above and below ground. The application of fertilizers has the potential to influence the patterns and processes associated with plant carbon inputs (Ali Shah et al., 2021). The results of this study showed that nitrogen and phosphorus applications increased SOC content. The primary reason may be attributed to the synergistic effect of nitrogen and phosphorus administration, resulting in a significant augmentation of both above-ground and root biomass in plants. Consequently, there is an amplified influx of plant litter and root secretions, thereby facilitating the process of plant carbon input (Shi et al., 2022). Under phosphorus application alone, SOC content tended to increase and then decrease with increasing phosphorus application, whereas under nitrogen and phosphorus combination conditions, SOC content tended to increase gradually. This may be due to the toxic effect of excessive phosphorus input to the soil, which exacerbates soil nitrogen limitation, inhibits plant growth activity and reduces SOC content (Ziehn et al., 2021). Phosphorus alone also exacerbates soil microbial nitrogen limitation and reduces soil microbial abundance, which in turn reduces SOC content (Liu et al., 2023). The application of both nitrogen and phosphorus can alleviate the nutrient limitation resulting from phosphorus alone, increase extracellular enzyme activity, accelerate the rate of decomposition of litter, and increase plant carbon input (Zhang et al., 2022a). Consequently, the sequestration of SOC is increased. However, it has also indicated that nitrogen and phosphorus inputs lead to a decrease in SOC content (Li et al., 2021b). The primary factor is the introduction of nitrogen and phosphorus inputs accelerates the decomposition of SOC by soil microorganisms, resulting in an imbalance between carbon decomposition and accumulation. Consequently, this alters the function of the soil carbon pool, shifting it from a carbon sink to a carbon source (Luo et al., 2019). The findings of this study indicate that the 0–20 cm soil layer exhibited the highest SOC content, while the 50–60 cm soil layer had the lowest SOC content. This disparity can be attributed to the accumulation of decomposed plant litter primarily in the surface layer, as well as the limited nutrient input with increasing soil depth. Additionally, the reduced activity of soil microorganisms in the lower layer contributed to a noticeable aggregation of SOC in the vertical distribution (Gao et al., 2021). The results of this study showed that the surface SOC content was higher under the N_1_P_2_ treatment compared to the other treatments. One aspect to consider is that the N_1_P_2_ treatment led to the greatest yield of alfalfa, resulting in an increased litter input into the soil, conversely, a balanced nitrogen to phosphorus ratio also enhanced the quality of the litter and promoted its decomposition (Tie et al., 2022). Consequently, this treatment exhibited the highest organic carbon content in the surface soil. This study also revealed that the SOC content was higher in 2021 compared to 2020, mainly due to the increase in SOC content with increasing fertilizer application time (Xu et al., 2021), while the periodic input of litter from alfalfa, a perennial forage grass, also increased SOC content (Wei et al., 2022). Therefore, the judicious application of nitrogen and phosphorus significantly affects the spatial and temporal distribution of SOC in alfalfa fields, effectively increasing SOC content and carbon sequestration potential.

In the present study, it was shown that SOC content was significantly correlated with the hay yield of alfalfa (R^2^ = 0.555). This is mainly due to the fact that fertilization plays a crucial role in enhancing plant biomass, resulting in an increase in carbon input in both plant roots and shoots. This, in turn, leads to an elevation in SOC (Xu et al., 2021). Moreover, the fluctuations in SOC levels impact the availability of soil nutrients, pH levels, and microbial activity, all of which ultimately influence crop yields (Waqas et al., 2020). In this study, the N_1_P_3_ treatment exhibited the highest SOC content, while the N_1_P_2_ treatment resulted in the highest hay yield. These findings align with previous research indicating that a sustained increase in SOC levels may lead to a slight decrease in crop yield (Lin et al., 2023). To maximize SOC sequestration and increase crop yield, the combined analysis showed that the N_1_P_2_ treatment provided a better balance between SOC content and alfalfa hay yield. Simultaneously, the SOC content is influenced by multiple factors such as management system, soil properties and natural conditions (Lal, 2020), and the best fertilizer application method should be determined according to the specific cropping system.

### 4.2 Effect of nitrogen and phosphorus application on soil active organic carbon in alfalfa fields

Soil active organic carbon is characterized by high activity, a rapid turnover rate, facile mineralization and decomposition, and the capacity to provide nutrients directly to crops. Its content is influenced by a combination of carbon input processes, such as plant and animal residues and root secretions, and carbon decomposition processes by microorganisms (He et al., 2022). The results of this study showed that the introduction of nitrogen and phosphorus significantly increased the active organic carbon content of the soil, probably because nitrogen and phosphorus addition influenced the decomposition of SOC by changing the diversity and abundance of microorganisms (Li et al., 2021a). In this research, it was found that soil EOC, DOC and POC contents exhibited their greatest values within the 0–20 cm soil layer when subjected to the same treatment. In addition, these contents exhibited a decreasing trend as the soil depth increased. This is mainly due to the phenomenon that the 0–20 cm soil layer has greater concentration of humus by litter and root secretions, which forms the fixation effect of organic colloids and makes the soil active organic carbon content higher. However, as the soil depth increases, the fixation effect rapidly diminishes (Juhos et al., 2021). MBC is the most active part of soil active organic carbon and is a highly responsive indicator of early soil changes, which originate from soil microorganisms (Patoine et al., 2022). In this research, the highest MBC content was exhibited only in the 10–20 cm soil layer. It can be attributed to the potential influence of climatic conditions on the biomass and activity of soil microorganisms (Jansson and Hofmockel, 2020). Furthermore, the 0–10 cm soil layer was subject to disturbances from the external environment, characterized by persistent dryness and low annual rainfall (Yao et al., 2018). Consequently, the soil microbial population in this layer was found to be relatively low. Simultaneously, the constraining elements for MBC content shifted with increasing soil depth. The study showed that microbial biomass in the upper soil layer was primarily influenced by nitrogen fertilizer and carbon sources. Additionally, the introduction of nitrogen and phosphorus resulted in enhanced nutrient effectiveness and litter input within the 10–20 cm layer, therefore leading to an increase in the abundance and variety of soil microorganisms (Tang et al., 2023). This outcome explains the highest MBC content under the N_1_P_2_ treatment. In contrast, subsoil microbial biomass was more influenced by soil factors and decreased with increasing soil depth (Sun et al., 2021). The content of soil active organic carbon was increased to varying degrees by the addition amount and proportion of nitrogen and phosphorus. Furthermore, the extent of this increase was shown to be influenced by the time of fertilizer application.

### 4.3 Effect of nitrogen and phosphorus application on soil organic carbon efficiency, sensitivity index and carbon management index

The usage of the ratio between soil active organic carbon and SOC provides a more accurate representation of the SOC trend (Qu et al., 2021). The stability of SOC may be inferred by examining the ratio of POC to SOC, likewise, the activity of SOC can be assessed by analyzing the ratio of MBC to SOC (Zhao et al., 2023). The relationship between the ratio of EOC to SOC and the decomposition capacity of SOC can be established. Additionally, the ratio of DOC to SOC may serve as an indicator of the extent of SOC loss (Tang et al., 2010). The results of this study showed that nitrogen and phosphorus inputs increased the ratio of POC to SOC. However, phosphorus application reduced the proportion of POC, in the 40–60 cm soil layer, mainly because the effect of phosphorus fertilization on soil carbon was primarily occurs within the 0–30 cm soil layer, while nitrogen fertilization could affect SOC content in the 0–120 cm soil layer (Zhong et al., 2015). Similarly, it proved that phosphorus fertilization can increase POC content but does not affect its spatial distribution (Hu et al., 2022). The proportion of POC to SOC exhibited the highest values, suggesting that POC made the greatest contribution to SOC. The structural equation modeling also revealed that POC had the highest cumulative effect on SOC. This is consistent with previous findings that POC plays a dominant role in the accumulation of SOC (Qu et al., 2021). Nitrogen and phosphorus inputs increased the ratio of EOC to SOC in the 0–10 cm soil layer, resulting in enhanced SOC activity in the 0–10 cm soil layer, while the ratio of EOC to SOC was lower in the 40–60 cm soil layer than in the N_0_P_0_ treatment, indicating that nitrogen and phosphorus inputs slowed the decomposition of SOC in the 40–60 cm soil layer, thereby increasing the potential for soil carbon sequestration. Similarly, the proportion of soil DOC to SOC in the 40–60 cm soil layer was also lower than in the N_0_P_0_ treatment, which further proved that the nitrogen and phosphorus input gave the subsoil a higher carbon sequestration potential (Tang et al., 2010). The sensitivity index is a measure of the soil reactive organic carbon component that exhibits the greatest responsiveness to various nitrogen and phosphorus treatments (Chaudhary et al., 2017). we revealed that the amount and rate of nitrogen and phosphorus additions did not have an equivalent influence on the sensitivity index of soil reactive organic carbon. In the alfalfa field, it was shown that MBC exhibited the greatest degree of responsiveness to nitrogen and phosphorus treatments within the 0–30 cm soil layer. Conversely, the POC was found to be highest in the 30–60 cm soil layer.

Compared with single indicators for evaluating soil fertility, such as SOC, the CMI index can provide a more sensitive evaluation of changes in SOC and serve as a valuable indicator for assessing soil fertility (Mandal et al., 2020; Rai et al., 2023). In this study, the application of nitrogen and phosphorus resulted in an increase in the CMI index within the 0–60 cm soil layer, except for the 20–30 cm soil layer under the N_0_P_1_ treatment and the 20–50 cm soil layer under the N_1_P_0_ treatment. This finding further supports the notion that fertilizer application contributes to the accumulation of SOC throughout all soil layers (Zhang et al., 2021). Additionally, the research revealed that L and LI index were higher in the 0–10 cm soil layer compared to the N_0_P_0_ treatment, while they were lower in the 40–60 cm soil layer under all fertilization treatments. This observation aligns with the previous discussion, indicating that nitrogen and phosphorus inputs enhance the decomposition of organic matter and nutrient cycling in the shallow soil layer (0–10 cm), while promoting the stability of the soil carbon pool in the deeper soil layer (Wang et al., 2015).

## 5 Conclusion

Nitrogen and phosphorus treatments increased SOC, MBC, POC, DOC, EOC and alfalfa hay yield, and this process increased with the duration of fertilization. However, the effect of different nitrogen and phosphorus treatments varied, with SOC, EOC, DOC, POC, MBC content and alfalfa hay yield all being highest under N_1_P_2_ or N_1_P_3_ treatments, increasing by 21.85%, 25.01%, 22.03%, 44.15%, 62.61% and 23.25%, respectively. Meanwhile, the CMI of different soil layers was sensitive to nitrogen and phosphorus additions, with the highest carbon pool management index under the N_1_P_2_ treatment. The comprehensive evaluation results indicated that the N_1_P_2_ treatment was the optimal fertilizer application. Structural equation analysis showed that MBC and POC content significantly influenced the accumulation of SOC. Compared with N_0_P_0_, the nitrogen and phosphorus treatments increased the efficiency of the soil active organic carbon fraction in the 0–40 cm soil layer but decreased the efficiency of the EOC and DOC in the 40–60 cm soil layer, indicating that the nitrogen and phosphorus treatments improved the soil fertility in the 0–40 cm soil layer and the soil carbon sequestration potential in the 40–60 cm soil layer. The highest MBC sensitivity index was found in the 0–30 cm soil layer, and the highest POC sensitivity index was found in the 30–60 cm soil layer. This indicates that as the depth of the soil layer increases, the indicator of SOC change from MBC to POC is changed by nitrogen and phosphorus. In conclusion, the application of appropriate nitrogen and phosphorus treatments in agroecosystems has proven to be a viable strategy for enhancing SOC levels and increasing alfalfa hay yield. Optimal outcomes have been seen when utilizing nitrogen at a rate of 120 kg·ha^-1^ and phosphorus at a rate of 100 kg·ha^-1^.

## Data availability statement

The original contributions presented in the study are included in the article/supplementary material. Further inquiries can be directed to the corresponding authors.

## Author contributions

KW: Conceptualization, Data curation, Formal analysis, Investigation, Methodology, Software, Validation, Visualization, Writing – original draft. JZ: Conceptualization, Data curation, Investigation, Writing – review & editing. YS: Conceptualization, Formal analysis, Software, Writing – review & editing. IL: Conceptualization, Writing – review & editing. CM: Conceptualization, Writing – review & editing. QZ: Conceptualization, Funding acquisition, Project administration, Resources, Supervision, Validation, Writing – review & editing.
